# Analysis of Dynamic Properties and Johnson–Cook Constitutive Relationship Concerning Polytetrafluoroethylene/Aluminum Granular Composite

**DOI:** 10.3390/ma18153615

**Published:** 2025-07-31

**Authors:** Fengyue Xu, Jiabo Li, Denghong Yang, Shaomin Luo

**Affiliations:** School of Aerospace Engineering, Guizhou Institute of Technology, Guiyang 550025, China; 15329276859@163.com (J.L.);

**Keywords:** impact-initiated energetic materials, PTFE/Al composite, mechanical properties, constitutive relation, dynamic compression

## Abstract

The polytetrafluoroethylene/aluminum (PTFE/Al) granular composite, a common formulation in impact-initiated energetic materials, undergoes mechanochemical coupling reactions under sufficiently strong dynamic loading. This investigation discusses the dynamic properties and the constitutive relationship of the PTFE/Al granular composite to provide a preliminary guide for the research on mechanical properties of a series of composite materials based on PTFE/Al as the matrix. Firstly, the 26.5Al-73.5PTFE (wt.%) composite specimens are prepared by preprocessing, mixing, molding, high-temperature sintering, and cooling. Then, the quasi-static compression and Hopkinson bar tests are performed to explore the mechanical properties of the PTFE/Al composite. Influences of the strain rate of loading on the yield stress, the ultimate strength, and the limited strain are also analyzed. Lastly, based on the experimental results, the material parameters in the Johnson–Cook constitutive model are obtained by the method of piecewise fitting to describe the stress–strain relation of the PTFE/Al composite. Combining the experimental details and the obtained material parameters, the numerical simulation of the dynamic compression of the PTFE/Al composite specimen is carried out by using the ANSYS/LS-DYNA platform. The results show that the computed stress–strain curves present a reasonable agreement with the experimental data. It should be declared that this research does not involve the energy release behavior of the 26.5Al-73.5PTFE (wt.%) reactive material because the material is not initiated within the strain rate range of the dynamic test in this paper.

## 1. Introduction

Impact-initiated energetic materials (also called reactive materials or energetic structural materials), which are different from traditional inert metal materials, will undergo a mechanical–chemical coupling reaction under sufficiently strong dynamic loadings (such as detonation wave, impact, etc.). This coupling reaction involving mechanics and chemistry is expected to greatly enhance the power of conventional warheads [1]. The PTFE/Al composite is a common formulation of impact-initiated energetic materials and has potential applications in the field of high damage. It can be used to fabricate low-density energetic fragments, the liners of shaped charges, the inner materials of penetrators, and so forth [1,2,3].

In recent decades, several researchers have dedicated significant attention to studies concerning the PTFE/Al composite to promote its application [4,5,6,7,8,9,10,11,12,13,14,15]. In 2003, Joshi et al. proposed a method to prepare PTFE/Al specimens by mixing, molding, and sintering. The weight ratio of Teflon to aluminum was about 3:1 [4]. Later, Nielson et al. improved the fabrication process by sintering the powder mixture in an inert gas environment and prepared the PTFE/Al/W energetic composite, in which the tungsten was used to increase the density [5]. Ames et al. proposed a method to characterize the energy release of the energetic composite by measuring the overpressure caused by the specimen in a closed chamber. The relation between the overpressure in the closed chamber and the released chemical energy of the energetic composite was also established [6,7]. In 2010, Chonowski designed a test device which was smaller and less expensive to perform studies on the energy release characteristics of energetic materials [8]. Raftenberg et al. conducted simulation modeling on the impact-induced deformation behavior of a cylindrical PTFE/Al composite specimen by using the Hopkinson bar and Taylor bar experiments [9]. Three kinds of impact experiments (including direct impact, indirect impact, and two-step impact) were performed to investigate the impact-ignited behavior of the PTFE/Al composites [10]. Mock et al. studied the influence of the particle size of aluminum powder on the impact-initiated characteristics of the PTFE/Al composite by using impact tests. Moreover, different impact-initiated behaviors of the PTFE/Al composite in vacuum and air were analyzed [11]. Ren et al. explored the influences of initial defects on the impact ignition of PTFE/Al reactive materials by using Hopkinson bar tests [12]. Based on experiments of explosive loading and the numerical simulation of the mesoscale behavior of the PTFE/Al composite, Tang et al. discussed the formation mechanism of hot spots in the PTFE/Al composite under shock loadings [13]. Xiao et al. estimated the velocity of the elastic wave and the slope in the equation of state (shock equation) for unreacted PTFE/Al materials by combining the Hopkinson bar tests and the fitting method [14]. Based on the Hopkinson bar tests, Jiang et al. analyzed the influence of porosity on the mechanical behavior and the impact ignition of the PTFE/Al energetic material with a high weight ratio of aluminum [15]. These studies indicated that the mechanical response and the impact-induced energy release were the two main directions in the study of PTFE/Al composites. The purpose of conducting mechanical response research is mainly to enhance mechanical properties, reveal failure mechanisms, construct constitutive relations, and so forth, while the main aim of studying impact-induced energy release is to explore the reaction threshold and conduct energy release analysis. Research on impact-induced energy release often requires the support of mechanical properties.

Studies on other types of impact-initiated energetic materials have also been carried out [16,17,18,19,20,21,22]. For example, Cai et al. analyzed influences of the particle size and the strain rate of loading on the mechanical behavior of the PTFE/Al/W composite [16,17]. Geng et al. explored the effects of the theoretical density, the molding pressure, and the drop height on the impact-induced initiation of the porous PTFE/Al/W reactive material by using drop weight tests [18]. The quasi-static and dynamic mechanical behaviors of the PTFE/Al/W reactive material were investigated by compression experiments. Moreover, the effect of the thermal softening and the forming behavior of the fiber network were analyzed by microscopic fractography [19]. Glavier et al. tested the burning rate of four kinds of nano-thermite mixtures (including Al/PTFE, Al/MoO_3_, Al/Bi_2_O_3_, and Al/CuO) by experiments. And the reaction rates of these nano-thermite mixtures were analyzed based on the pressure signals [20]. Wang et al. introduced a ductile coating of Ni to the tungsten–zirconium alloy to improve the damage potential of the reactive projectile [21]. Zhou et al. explored the mechanical properties and the impact-induced reaction of PTFE/Al/CuO reactive materials by the combined method of scanning electron microscopy, quasi-static compression, the Hopkinson bar test, and the drop weight test [22].

These research works have significant reference value for the enhancement in mechanical properties and the application of impact-initiated energetic materials. However, systematic studies on the mechanical properties and the constitutive relationships of a certain series of impact-initiated energetic materials remain insufficient. For instance, PTFE/Al is a typical formulation of impact-initiated energetic materials. Based on this formulation, a series of impact-initiated energetic materials can be fabricated by adding metals or metal oxides.

According to the chemical reaction equation $3C_{2}F_{4}+4Al=4AlF_{3}+6C$, zero oxygen balance can be achieved in the equation when the formulation is 26.5Al-73.5PTFE (wt.%), which is conducive to the chemical reaction between the components. Moreover, many researchers have prepared many new composite materials based on this formulation, such as the PTFE/Al/W composite material in reference [18] (including 51.0PTFE/18.4Al/30.6W, 21.4PTFE/7.7Al/70.9W, etc.), the PTFE/Al/CuO composite material in reference [22], and so forth. In this paper, a series of nine high-strain-rate compression tests is performed using a split Hopkinson bar to study the dynamic response and constitutive relation of 26.5Al-73.5PTFE (wt.%) granular composites. This research has preliminary guiding significance for the systematic study of the mechanical properties of a series of composite materials based on PTFE/Al as the matrix. Moreover, the results can provide a guide for the study of the modeling of the dynamic constitutive relation, the optimization of simulation methods, and the characterization of mechanical properties. It should be declared that within the strain rate range of the dynamic test conducted in this paper, the 26.5Al-73.5PTFE (wt.%) reactive material is not initiated in a chemical reaction. Therefore, this research does not involve the energy release behavior of the material.

## 2. Experimental Details

### 2.1. Preparation of Specimens

The preparation process of the PTFE/Al composite specimens included mixing, molding, sintering at high temperature, and cooling. The polytetrafluoroethylene powder and the aluminum powder were supplied by Guizhou Guitiancheng Technology Co., Ltd. (Guiyang, China). The weight ratio of polytetrafluoroethylene to aluminum was 73.5:26.5, and they were weighed before mixing. The powder mixture of PTFE and Al was then prepared by a mixer. The diameters of PTFE and Al were 100 nm and 5 μm, respectively.

**Figure 1:**
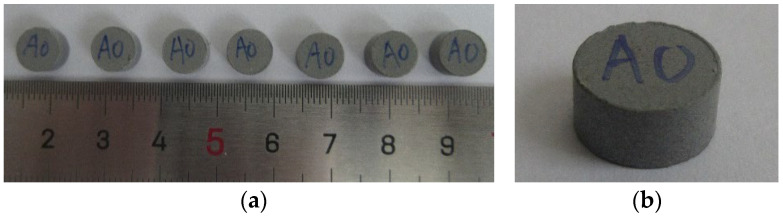
Photographs of the 26.5Al-73.5PTFE (wt.%) composite specimens: (**a**) distant view of the specimens and (**b**) close-up view of a cylindrical specimen.

**Figure 2:**
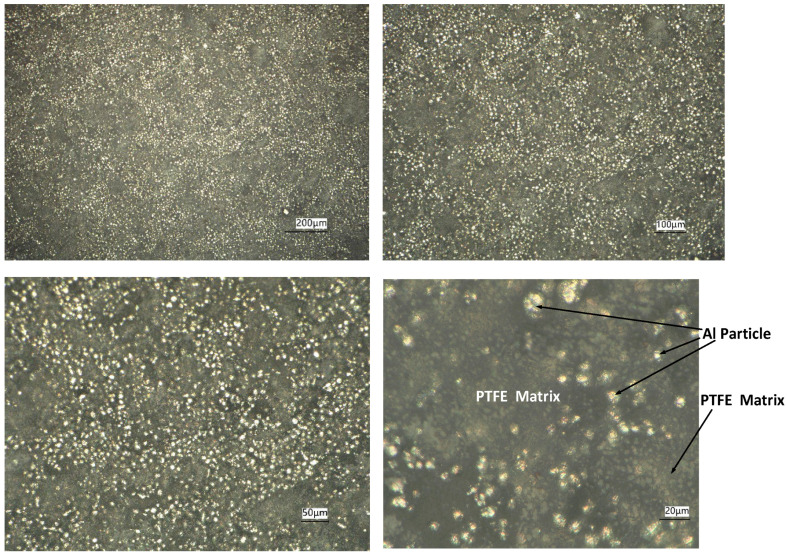
Microstructure of a cylindrical specimen on the bottom surface with different views.

The maximum value of the molding pressure was 200 MPa. And the molding pressure needed to be maintained for five minutes at the maximum value before unloading. The change in temperature in the sintering and cooling process needed to be strictly controlled. Firstly, the temperature in the sintering oven gradually increased to 380 °C at a rising rate of 50 °C/h and was kept at 380 °C for 4 h. Then, the temperature decreased to 310 °C at a rate of 50 °C/h and was kept at 310 °C for 2 h. Lastly, the temperature decreased to room temperature at a rate of 50 °C/h. The actual density of these specimens was 2.28 g/cm^3^. The theoretical density of the 26.5Al-73.5PTFE (wt.%) composite was 2.32 g/cm^3^. So, the value of porosity that could be calculated was 1.8%. Some of the cylindrical specimens of the 26.5Al-73.5PTFE (wt.%) composite are shown in Figure 1. The average size of the cylindrical specimens was Φ7.86 × 4.33 mm. A VHX-7000 digital microscope system (Keyence Corporation, Osaka, Japan) was used to investigate the microstructure of the specimens, as shown in Figure 2. The microstructure showed that the PTFE matrix was distributed as a continuous network structure, forming a framework structure that went through the entire specimen. This framework structure provided the basic mechanical support for the particle composite material. The Al particles were evenly embedded in the PTFE matrix, and the phenomenon of Al particle agglomeration was observed in some areas. During deformation, the force chain effect between the Al particles was expected to enhance the load-bearing capacity of the specimen.

### 2.2. Experimental Method of Quasi-Static Compression

**Figure 3:**
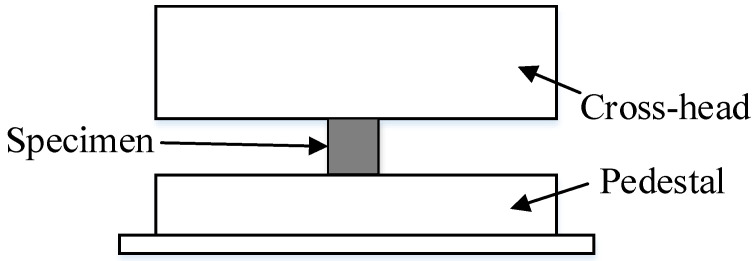
Schematic of quasi-static compression.

The quasi-static compression experiments were carried out by using the WDT series universal material testing machine (Zhongji Test Equipment Co., Ltd., Changchun, China), and the experimental schematic is shown in Figure 3. In a test, a cylindrical specimen of the 26.5Al-73.5PTFE (wt.%) composite was positioned at the center of the pedestal. Three specimens were used in quasi-static compression. The experiments were conducted under stroke control at a strain rate of loading of around 0.001 s^−1^. The friction on the contact surface between the specimen and the metal plate was ignored. It was also assumed that the deformation of the specimen during compression was uniform and continuous. Then, true stress and strain could be computed by using the compression force *P* based on Equations (1)–(3) [22].

(1) $$\sigma_{t}=\frac{P}{A_{0}}\left(1-\varepsilon_{e}\right)$$

(2) $$\varepsilon_{t}=\ln\frac{1}{1-\varepsilon_{e}}$$

(3) $$\varepsilon_{e}=\frac{\left(H_{0}-H\right)}{H_{0}}$$

where the variable *σ*_t_ denotes the true stress in the specimen. The variable *ε*_t_ denotes the true strain in the specimen. The quantities *A*_0_, $\varepsilon_{e}$, *H*, and *H*_0_ denote the initial cross-sectional area of the specimen, the engineering strain, the height of the deformed specimen, and the initial height of the specimen, respectively.

### 2.3. Experimental Method of Hopkinson Bar Tests

**Figure 4:**
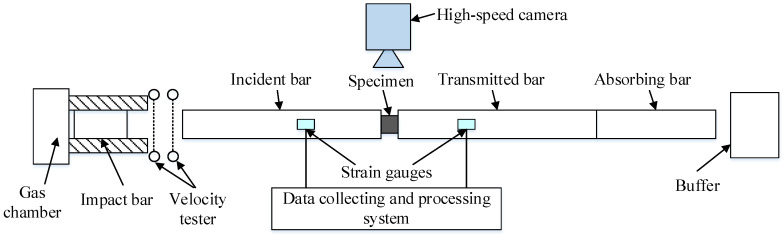
Schematic of Hopkinson bar tests.

In order to explore the dynamic properties of the 26.5Al-73.5PTFE (wt.%) composite specimens, Hopkinson pressure bar tests were performed. As shown in Figure 4, the test system consisted of a gas chamber, an impact bar, an incident bar, a transmitted bar, an absorbing bar, a buffer, a velocity tester, strain gauges, a signal acquisition system, and so forth. Based on the theoretical analysis of the one-dimensional stress wave, the stress $\sigma_{s}(t)$, the strain $\varepsilon_{s}(t)$, and the strain rate ${\dot{\varepsilon }}_{s}(t)$ in the specimen could be written as [23]

(4) $${\;\sigma }_{s}(t)=EA\varepsilon_{T}(t)/A_{s}$$

(5) $$\varepsilon_{s}(t)=-2C_{0}/L_{s}\int_{0}^{t}\varepsilon_{R}(t)dt$$

(6) $${\dot{\varepsilon }}_{s}(t)=-2C_{0}\varepsilon_{R}(t)/L_{s}$$

where the variable *C*_0_ denotes the velocity of the elastic wave in the metal bars. The variable *E* denotes the elasticity modulus of the metal bars. The value of variable *E* is 70 GPa in the tests. The variable $\varepsilon_{R}(t)$ denotes the reflected strain in the incident bar. The variable $\varepsilon_{T}(t)$ denotes the transmission strain in the transmitted bar. The quantities *A*, *A*_s_, *L*_s_, and *t* denote the cross-sectional area of the metal bars, the cross-sectional area of the specimen, the length of the specimen, and the time, respectively. It was also assumed that the deformation of the specimen during compression was uniform and continuous. The true stress $\sigma_{t}\left(t\right)$ and the true strain $\varepsilon_{t}\left(t\right)$ in the compressed specimen can be computed by using Equations (7) and (8) [23].

(7) $$\sigma_{t}\left(t\right)=\sigma_{s}\left(t\right)\cdot (1-\varepsilon_{s}\left(t\right))$$

(8) $$\varepsilon_{t}\left(t\right)=ln(1-\varepsilon_{s}\left(t\right))$$

**Figure 5:**
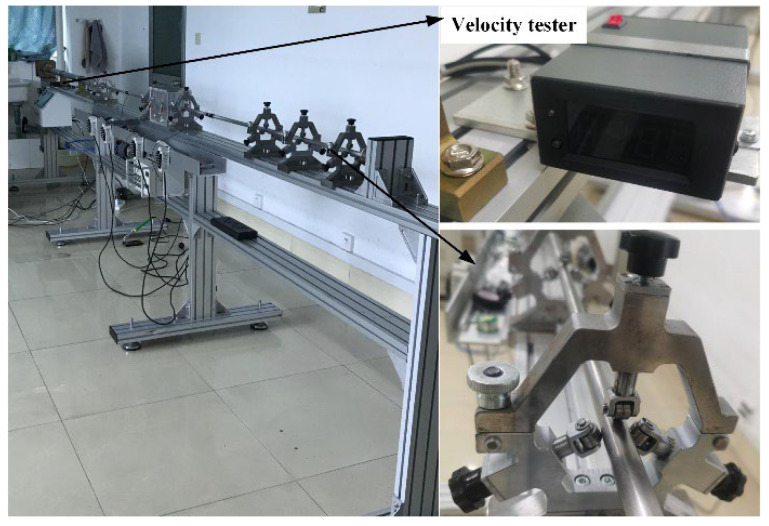
Photographs of experimental setup in the SHPB tests.

Figure 5 shows typical photographs of the experimental devices used in Hopkinson pressure bar tests. The tests were conducted at room temperature. Nine specimens were used to investigate the influence of the strain rate on the dynamic properties of the PTFE/Al composite. In a test, the strain rate could be controlled based on the approximation relation of the length of a specimen, the impact velocity, and the strain rate. The speed of the impact bar was adjusted by controlling the pressure in the gas chamber. This equipment had a maximum adjustable pressure range in the gas chamber of 0 to 0.75 MPa, which could meet the requirements of these tests. The voltage of the electricity bridge in the data acquisition system was 2 V. The magnification of the dynamic strain amplifier was set as 1000. The frequency of the signal acquisition was selected as 20 MHz. The diameter of the 7075 T6 aluminum bars used in the tests was 12 mm. The length of the impact bar was 200 mm, and the length of the incident bar and the transmitted bar was 1245 mm. The type of strain gauge attached to the incident bar and transmitted bar was BE120-3AA. And the sensitivity coefficient of the strain gauges was 2.08 ± 1%. A high-speed camera was used to record the fragmentation process of specimens during the tests, and the frame rate of the high-speed camera was 10^5^ frames per s.

## 3. Results and Discussion

### 3.1. Experimental Results

**Figure 6:**
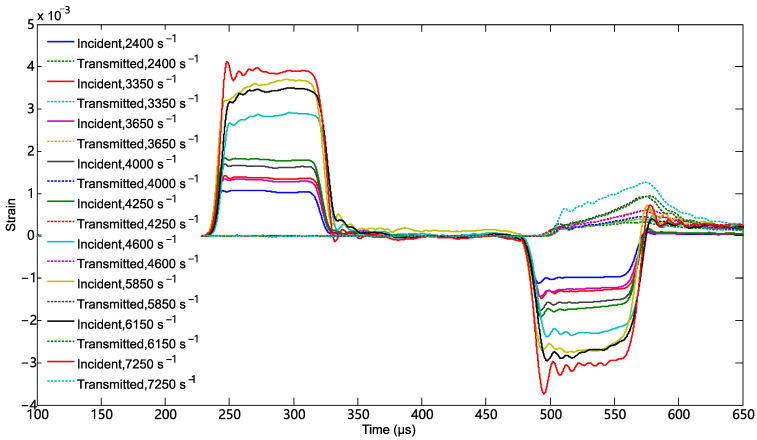
The signal curves of strain vs. time in incident bar and transmitted bar at different strain rates.

**Figure 7:**
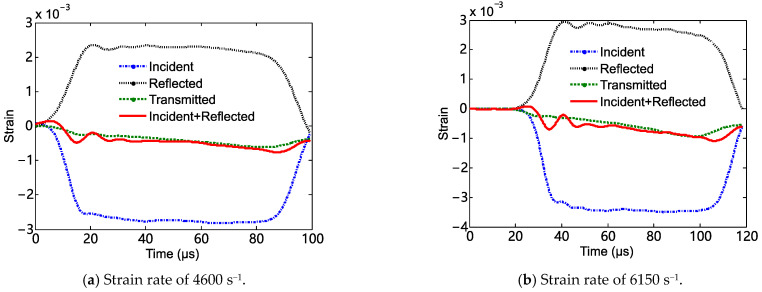
Comparisons of the incident wave, the reflected wave, and the transmitted wave.

The curves of the strain signal versus time in dynamic tests are shown Figure 6. The incident strain signal, the reflected strain signal, and the transmitted strain signal correspond to the top-left, the bottom-right, and the top-right pulses, respectively. When the impact bar impacted the incident bar, a shock wave propagated forward along the incident bar. Once this shock wave reached the interface between the specimen and the incident bar, a reflected wave and a transmitted wave were formed. The strain signals caused by the initial shock wave and the reflected wave were the incident strain signal and the reflected strain signal in Figure 6, respectively. The strain signal measured in the transmitted bar was caused by the transmitted wave. Figure 7 shows comparisons of the incident wave, the reflected wave, and the transmitted wave at typical strain rates. The transmitted wave and the superposition of the incident and reflected wave show reasonable agreement, indicating a good stress equilibrium in the specimens. Then, the true stress–strain curves of the composite in dynamic tests could be obtained by using the two-wave method.

**Figure 8:**
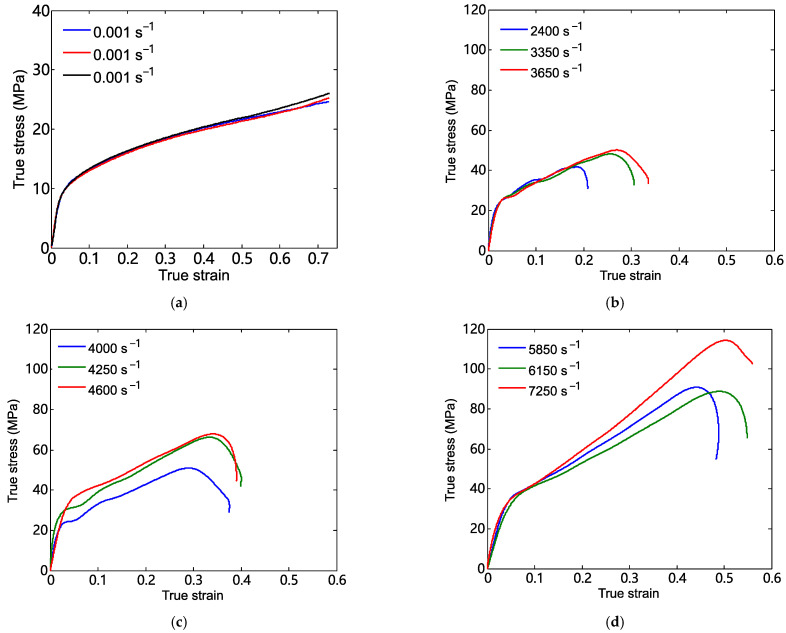
True stress–true strain curves of the 26.5Al-73.5PTFE (wt.%) composite specimens at (**a**) a strain rate of 0.001 s^−1^, (**b**) strain rates of 2400 s^−1^, 3350 s^−1^, and 3650 s^−1^, (**c**) strain rates of 4000 s^−1^, 4250 s^−1^, and 4600 s^−1^, and (**d**) strain rates of 5850 s^−1^, 6150 s^−1^, and 7250 s^−1^.

**Figure 9:**
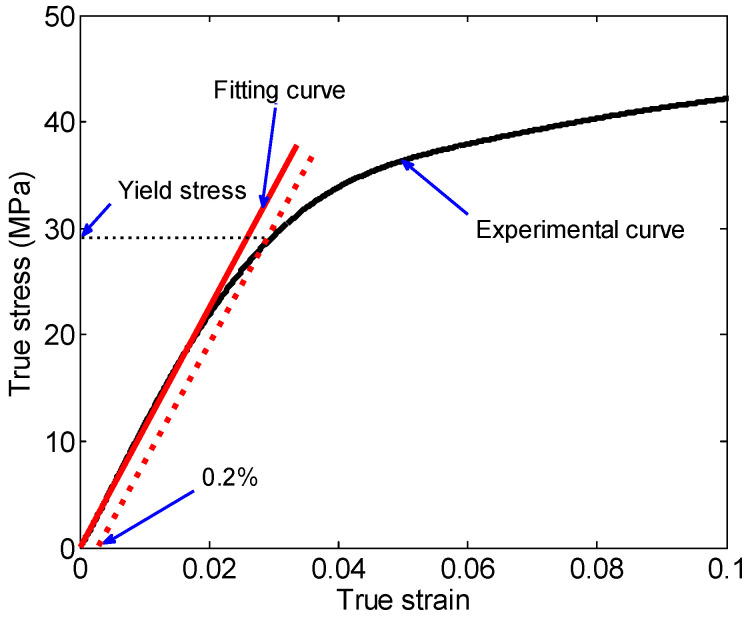
Schematic of the method for determining the yield stress.

Figure 8a gives the true stress–strain curves for three tests of the 26.5Al-73.5PTFE (wt.%) composite specimens at a strain rate of 0.001 s^−1^. Figure 8a shows that the three stress–strain curves were almost overlapped, and the standard deviation of the true stress at a strain of 0.7 was 0.5991. It was indicated that the experimental results in this investigation of the 26.5Al-73.5PTFE (wt.%) composite specimens were reliable and repeatable. The average yield stress of the specimen under quasi-static loading was about 8.0 MPa, which was obtained based on the method shown in Figure 9.

The true stress–strain curves of the 26.5Al-73.5PTFE (wt.%) composite specimens under dynamic loadings at different strain rates are shown in Figure 8b–d. It should be noted that the reported values for the dynamic tests are representative results. The results showed that the true stress in the specimen was approximately linearly related to the true strain before the true stress increased to the yield stress. After exceeding the yield stress, the value of the true stress in the specimen still presented an increasing trend with an increase in the true strain. But the increasing rate was much less than that in the beginning. Lastly, when the true stress exceeded the ultimate strength of the specimen, the true stress in the specimen decreased sharply with the increase in the true strain.

By analyzing the stress–strain curves and applying the yield stress determination method illustrated in Figure 9, the statistical results of compression experiments presented in Table 1 were obtained. The limited strain denoted the value of the true strain when the true stress reached the ultimate strength. Table 1 shows that the yield stress (including engineering yield stress), the ultimate strength (including engineering ultimate strength), and the limited strain presented an increasing trend with the increase in the strain rate of loading. Specifically, with the strain rate gradually increasing from 2400 s^−1^ to 7250 s^−1^, the yield stress increased from 17.28 MPa to 31.36 MPa, and the ultimate strength increased from 41.7 MPa to 114.31 MPa. Moreover, the limited strain ranged from 0.1872 to 0.5037.

It has been reported by Jiang et al. [15] that the yield strength and the compressive strength of PTFE/Al (porosity 1.2%) exhibit a strain rate effect, which is consistent with our findings in this paper. However, the mass ratio of the PTFE/Al composite studied by Jiang is 50:50 [15]. The dynamic tests in Refs. [20,22] have also shown that the strain rate of loading plays a vital role in the mechanical behavior (e.g., compressive strength) of the reactive materials (PTFE/Al/CuO, PTFE/Al/W).

**Figure 10:**
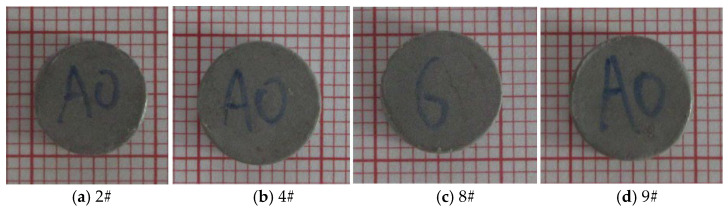
Photographs of the deformed specimens after dynamic testing with different strain rates. (**a**) The strain rate was 2400 s^−1^, and the diameter was about 9.10 mm. (**b**) The strain rate was 3650 s^−1^, and the diameter was about 9.71 mm. (**c**) The strain rate was 5850 s^−1^, and the diameter was about 9.67 mm. (**d**) The strain rate was 6150 s^−1^, and the diameter was about 10.00 mm.

**Figure 11:**
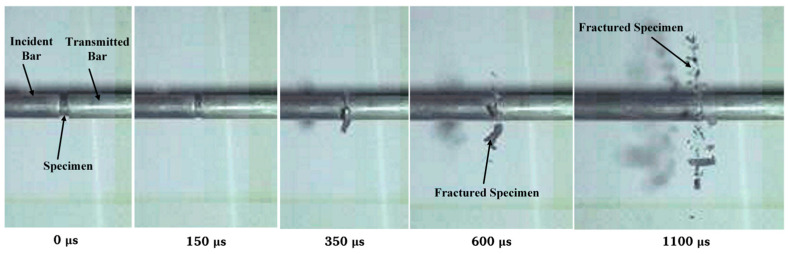
High-speed video frames of the fragmentation process at a strain rate of 7250 s^−1^. The compressed specimen fractured into fragments rather than only undergoing plastic deformation.

Typical photographs of the deformed specimens after dynamic testing are shown in Figure 10, in which the minimum size of the grid on the coordinate paper is 1 mm. The initial diameter of the specimen before testing was 7.86 mm. After dynamic compression, plastic deformation was obviously observed on the specimen. When the strain rate of loading was 2400 s^−1^, the diameter of the deformed specimen was about 9.10 mm, which is 1.16 times as large as the initial diameter. When the strain rate of loading was 6150 s^−1^, the diameter of the deformed specimen was about 10 mm, which is 1.27 times as large as the initial diameter. However, no obvious cracks were found on the surface of the deformed specimen by naked-eye observation in most tests. When the strain rate of dynamic loading increased to 7250 s^−1^, the specimen fractured into lots of fragments during the test, and typical high-speed video frames of the fragmentation process are shown in Figure 11.

**Figure 12:**
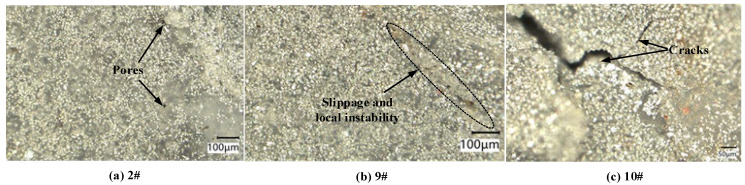
Microstructure of some compressed specimens. (**a**) Pores were observed at a strain rate of 2400 s^−1^. (**b**) Slippage zone was observed at a strain rate of 6150 s^−1^. (**c**) Many cracks were observed on the recovered residual fragment at a strain rate of 7250 s^−1^.

To understand more about the failure behavior of the specimens under dynamic compression, a VHX-7000 digital microscope system was used to investigate the microstructure of the compressed specimens and fragments, as shown in Figure 12. When the strain rate was 2400 s^−1^, many pores were found on the surface of specimen, and the number was greater than that before compression. When the strain rate was 6150 s^−1^, a distinct slippage zone was observed on the surface, which indicated that the PTFE matrix was likely to have undergone softening. When the strain rate increased to 7250 s^−1^, the compressed specimen fractured into fragments (see Figure 11), and many cracks were observed on the recovered residual fragment.

It has been observed by Cai et al. [16] that most of the plastic deformation takes place in the “soft” PTFE, and the W particles remain virtually undeformed. In unconfined PTFE/Al/W specimens, failure by cracking and shear localization immediately follows maximum stress [16]. Herbold et al. [17] reported that a relatively weak PTFE polymer matrix allows the strength and fracture mode of the PTFE/Al/W composite to be governed by the granular behavior of metal particles.

### 3.2. Simplified Johnson–Cook Constitutive Relationship

The Johnson–Cook constitutive model is used to describe the stress–strain relation of the PTFE/Al composite, which is not initiated in a chemical reaction in the tests. The stress–strain relation of the specimen under dynamic compression can be expressed as [24,25]

(9) $$\sigma =\left(A+B\cdot \varepsilon_{p}^{n}\right)\left[1+C\cdot ln\left(\frac{\dot{\varepsilon }}{\dot{\varepsilon_{0}}}\right)\right]\left(1-{\left(\frac{T-T_{r}}{T_{m}-T_{r}}\right)}^{m}\right)$$

where the variable σ denotes stress. The variable *ε*_p_ denotes the effective plastic strain. The variable $\dot{\varepsilon }$ denotes the plastic strain rate. The variable ${\dot{\varepsilon }}_{0}$ denotes the reference strain rate, and the value is given as $\dot{\varepsilon_{0}}=10^{-3}s^{-1}$. The variables A, B, C, n, and m denote the material constants. The variable *T* denotes the temperature. The variable $T_{r}$ denotes the reference temperature. The variable $T_{m}$ denotes the melting point of the material.

If the self-heating of materials during compression is not considered, the “m” parameter in Equation (9) can be ignored. Equation (9) can be simplified as

(10) $$\sigma =\left(A+B\cdot \varepsilon_{p}^{n}\right)\left[1+C\cdot ln\left(\frac{\dot{\varepsilon }}{\dot{\varepsilon_{0}}}\right)\right]$$

Furthermore, Equation (10) can be simplified as Equation (11) when the PTFE/Al specimen is in the state of critical yield, in which the variable *ε*_p_ is equal to zero.

(11) $$\sigma_{y}=A\left[1+C\cdot ln\left(\frac{\dot{\varepsilon }}{\dot{\varepsilon_{0}}}\right)\right]$$

Equation (11) shows that the yield stress of the specimen is linearly related to the logarithm of the strain rate. The computation method for estimating stress by Equation (11) does not consider the thermal/chemical coupling of reactive materials during compression. This point is likely to influence the estimation of true stress, especially when the strain rate is sufficiently high and the material may be initiated.

**Figure 13:**
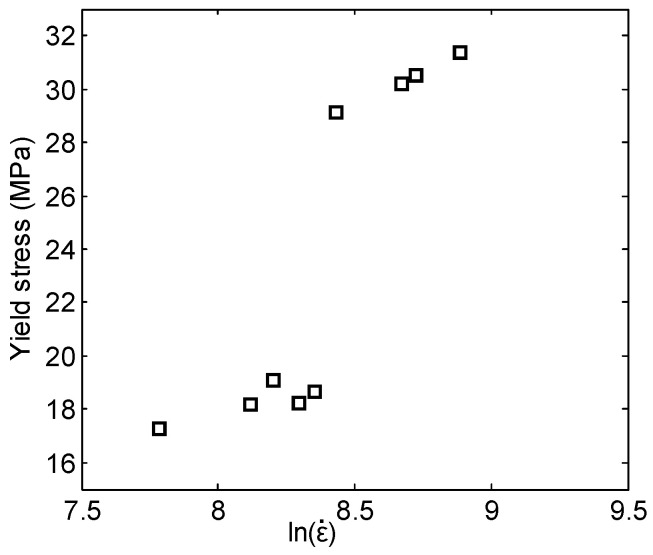
Relationship between the yield stress and the logarithm of the strain rate.

Based on the experimental results listed in Table 1, the relationship between the yield stress of the 26.5Al-73.5PTFE (wt.%) composite and the logarithm of the strain rate was obtained, as shown in Figure 13. The yield stress of the composite showed a gradual increase with the increase in the logarithm of the strain rate. Moreover, when the strain rate of loading increased from 4250 s^−1^ to 4600 s^−1^, the increment in the yield stress was much greater than that in other cases. In other words, there is a strain rate transition point at which the sensitivity of the yield stress to the strain rate changes greatly. So, the piecewise fitting method could be used to describe the relations between the yield stress and the logarithm of the strain rate in this study to achieve good matching. The critical value of the strain rate was chosen as 4600 s^−1^.

The method of determining the values of the parameters A, B, C, and n in the Johnson–Cook model can be described as follows: Firstly, the strain rate of loading was set as 0.001 s^−1^ in Equation (11), and the value of parameter A could be obtained. Then, based on Equation (11) and the experimental data shown in Figure 11, the values of parameter C could be obtained by piecewise fitting the relation between the yield stress and the logarithm of the strain rate. Lastly, the power function was used to fit the stress–strain curves when the strain rates of loading were 3650 s^−1^ and 5850 s^−1^, respectively. And the parameters n and B could be computed based on Equation (10). The values of the parameters A, B, C, and n of the 26.5Al-73.5PTFE (wt.%) composite in the Johnson–Cook model are listed in Table 2. The parameters A, B, n, and C are the yield stress at a strain rate of 0.001 s^−1^, the constant characterizing the material’s strain hardening, the index characterizing strain hardening, and the coefficient characterizing strain rate hardening, respectively.

**Figure 14:**
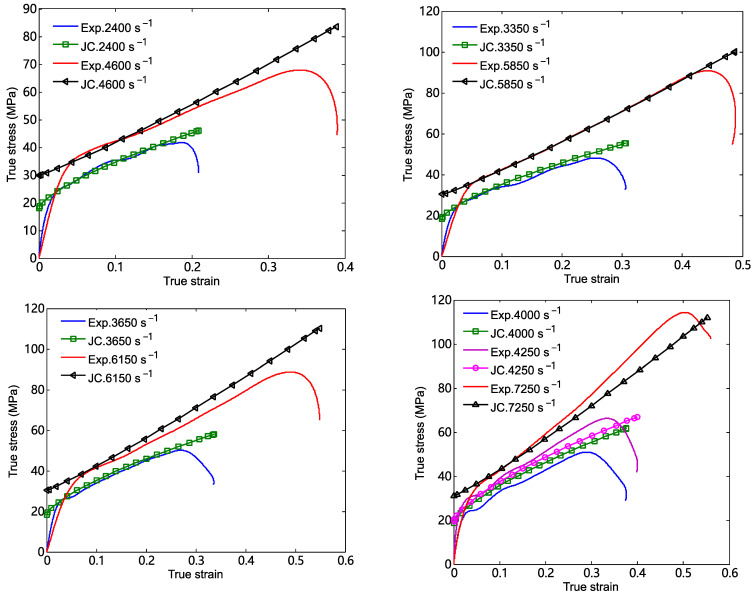
Comparisons between the computed results of simplified Johnson–Cook model and the experimental curves at different strain rates.

The simplified Johnson–Cook model can be obtained by substituting the parameters listed in Table 2 into Equation (10). This model can be used to describe the true stress–true strain relation of the 26.5Al-73.5PTFE (wt.%) composite under dynamic compression. According to the experimental details and the parameters of the specimens, comparisons between the computed results of the simplified Johnson–Cook model and the experimental curves are presented, as shown in Figure 14. It should be pointed out that the computed results were mainly used to compare with the stress–strain curves at the approximately linear stage after the yield stress. Figure 13 shows that the stress–strain curve computed by the simplified Johnson–Cook model presented a reasonable agreement with the experimental curves of the specimen in the plastic deformation stage. It was indicated that the constitutive parameters in Table 2 were applicable to describe the stress–strain relation of the 26.5Al-73.5PTFE (wt.%) composite when the strain rate of loading ranged from 2400 s^−1^ to 7250 s^−1^.

### 3.3. Numerical Simulation of Dynamic Compression of PTFE/Al Composite

**Figure 15:**
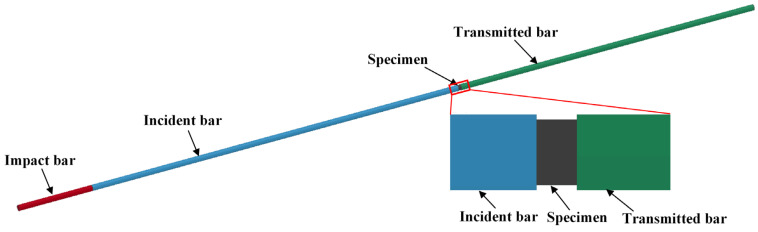
Simulated model of dynamic behavior of PTFE/Al composite specimen.

To reduce the cost and shorten the time of research promoting the application of the PTFE/Al composite, it is of importance to perform research on the method of numerical simulation. Based on the ANSYS/LS-DYNA platform, a simulated model for investigating the dynamic behavior of the PTFE/Al composite specimen was established, as shown in Figure 15. In the simulated model, the diameter of the metal bars (including the impact bar, the incident bar, the transmitted bar, and the absorbing bar) was 12 mm, which showed agreement with the tests. The elastic model was selected to describe the metal bars, which had a density of 2.7 g/cm^3^ and an elastic modulus of 70 GPa. Considering the specimen size in the experiment and the convenience of calculation, the diameter and the length of the 26.5Al-73.5PTFE (wt.%) composite specimen were chosen as 8 mm and 4 mm, respectively. The Poisson’s ratio of the PTFE/Al material in simulation was set as 0.34. In the simulation, the simplified Johnson–Cook model was used to describe the stress–strain relation of the 26.5Al-73.5PTFE (wt.%) composite.

**Figure 16:**
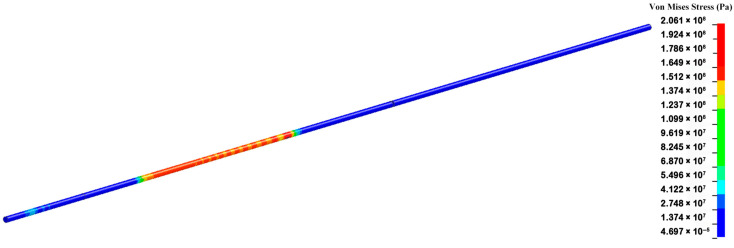
Typical simulation process of dynamic compression.

The simulated model was meshed by using the SOLID164 grid, which is an eight-node hexahedron element. Due to the one-dimensional mechanics process of the SHPB experiment in the design, the axial mesh in the metal bars could be simplified appropriately. The minimum size of the grid element in the specimen was 0.05 mm, and the minimum size of the grid element in the metal bars was 0.4 mm. During the dynamic compression, the impact bar impacted the incident bar at a given speed. Then, two shock waves produced at the time of the impact propagated forward into the incident bar and backward into the impact bar. The shock wave propagated forward along the incident bar to load on the specimen, which was in the middle of the incident bar and the transmitted bar. The typical simulation process of dynamic compression is shown in Figure 16. The time when the shock wave arrived at the specimen was 0 μs.

**Figure 17:**
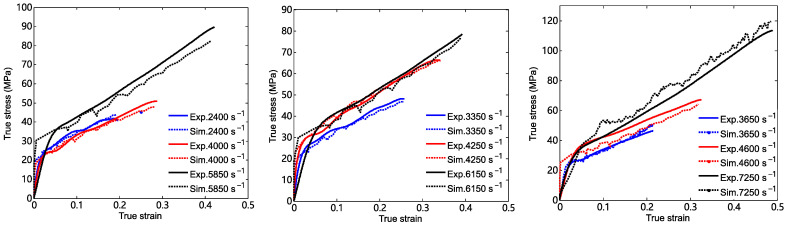
Comparisons of true stress–strain curves between the simulated results and the experimental results at different strain rates.

Based on the experimental parameters in the dynamic tests, comparisons of the true stress–strain curves between the simulated results and the experimental results were obtained, as shown in Figure 17. It shows that the stress–strain curves computed by the numerical simulation show a relatively reasonable agreement with those of the experiments when the stress is greater than the yield stress. In particular, the maximum difference between the values of simulated stress and the values of experimental stress at the same strain is about 8% when the strain rate of loading is 3650 s^−1^. In other words, the fitting parameters in Table 2 are reliable when the Johnson–Cook model is used to characterize the dynamic constitutive relationship of the 26.5Al-73.5PTFE (wt.%) composite. These parameters can be used for other numerical simulations of this PTFE/Al composite. Fluctuations in the stress–strain dependencies within the numerical implementation of the problem are observed. The meshing of the finite element may be one of the causes of stress–strain fluctuations.

## 4. Conclusions

A combined method of experiment and simulation was performed to investigate the mechanical properties and the constitutive relation of the 26.5Al-73.5PTFE (wt.%) composite. The results showed that the strain rate of loading had a significant influence on the mechanical properties. The yield stress, the ultimate strength, and the limited strain presented an increasing trend with the increase in the strain rate of loading. Specifically, with the strain rate of loading gradually increasing from 2400 s^−1^ to 7250 s^−1^, the yield stress, the ultimate strength, and the limited strain increased from 17.28 to 31.36 MPa, from 41.70 to 114.31 MPa, and from 0.1872 to 0.5037, respectively. Based on the experimental results and the Johnson–Cook model, a simplified constitutive relationship was obtained, in which the material parameters A, B, n, and C of the 26.5Al-73.5PTFE (wt.%) composite were determined by piecewise fitting. Moreover, the comparisons of the stress–strain curves showed that the computed results presented reasonable agreement with the experimental data when the strain rate of loading ranged from 2400 s^−1^ to 7250 s^−1^.

The PTFE-Al composite system integrates the excellent self-lubricating properties of PTFE with the high thermal conductivity of aluminum, offering significant potential for multifunctional applications. For example, the application of a thin-plate PTFE/Al composite in Whipple shielding structures is anticipated to significantly improve spacecraft protection against hypervelocity space debris impacts. Concurrently, the formulation of aluminum–PTFE hybrid coatings presents a viable approach for enhancing the corrosion resistance of critical aircraft components, particularly titanium alloy fasteners, the skin, and the shaft. Based on the 26.5Al-73.5PTFE (wt.%) composite, a series of reactive materials can be fabricated by adding metals or metal oxides. These reactive materials have potential applications in engineering fields. This paper focuses on the dynamic properties and the dynamic constitutive relation of the 26.5Al-73.5PTFE (wt.%) granular composite, providing guiding significance for the application of PTFE/Al and its derivative composite materials.

## Figures and Tables

**Table 1:** Experimental statistical results.

Test No.	Strain Rate (s^−1^)	Yield Stress (MPa)	Ultimate Strength (MPa)	Limited Strain	Engineering Yield Stress (MPa)	Engineering Ultimate Strength (MPa)
1-1#	0.001	8.0	--	--	8.02	--
1-2#	0.001		--	--		--
1-3#	0.001		--	--		--
2#	2400	17.28	41.70	0.1872	17.31	50.28
3#	3350	18.16	53.53	0.2565	18.20	69.18
4#	3650	19.07	55.70	0.2698	19.11	72.95
5#	4000	18.25	59.45	0.2904	18.29	79.48
6#	4250	18.64	66.27	0.3348	18.68	92.62
7#	4600	29.12	67.91	0.3416	29.18	95.56
8#	5850	30.22	90.79	0.4426	30.28	141.34
9#	6150	30.52	92.97	0.4904	30.58	151.82
10#	7250	31.36	114.31	0.5037	31.42	189.16

**Table 2:** Fitting parameters of the 26.5Al-73.5PTFE (wt.%) composite.

The Value of Strain Rate	A (MPa)	B (MPa)	n	C
$\dot{\varepsilon }<4600\;s^{-1}$	8.0	36.89	0.7005	0.087
$\dot{\varepsilon }\geq 4600\;s^{-1}$	8.0	40.62	1.117	0.182

## Data Availability

The original contributions presented in this study are included in the article. Further inquiries can be directed to the corresponding author.

## Abbreviations

**Symbol**	**Meaning**
*σ*_t_	True stress in the specimen
*ε*_t_	True strain in the specimen
*A*_0_	Initial cross-sectional area of specimen
$\varepsilon_{e}$	Engineering strain
*H*	Height of deformed specimen
*H*_0_	Initial height of specimen
*C*_0_	Velocity of elastic wave in the metal bars
*E*	Elasticity modulus of the metal bars
$\varepsilon_{R}(t)$	Reflected strain in the incident bar
$\varepsilon_{T}(t)$	Transmission strain in the transmitted bar
*A*	Cross-sectional area of the metal bars
*T*	Temperature
$T_{r}$	Reference temperature
*A*_s_	Cross-sectional area of specimen
*L*_s_	Length of specimen
*t*	Time
$\sigma_{s}(t)$	Stress in the specimen under dynamic compression
$\varepsilon_{s}(t)$	Strain in the specimen under dynamic compression
${\dot{\varepsilon }}_{s}(t)$	Strain rate of the specimen under dynamic compression
σ	Stress
*ε*_p_	Effective plastic strain
$\dot{\varepsilon }$	Plastic strain rate
${\dot{\varepsilon }}_{0}$	Reference strain rate
A, B, C, n, and m	Material constants
$T_{m}$	Melting point of material
$\sigma_{y}$	Yield stress

## References

[1] Wang H.F., Xiang J.A. (2023). Progress in reactive materials and their applications (in Chinese). Sci. Sin. Technol..

[2] DE Technologies Inc (2006). Reactive Fragment Warhead for Enhanced Neutralization of Mortar, Rocket, & Missile Threats ONR-SRIR: N04-903. https://www.detk.com.

[3] Cai Y., Feng X.Y., He C., Zhang S., Li S.K., Liu J.X. (2024). A 7.62 mm energetic bullet filled with PTFE-Mg-based reactive materials for anti-drone application. J. Mater. Res. Technol..

[4] Vasant S.J., Waldorf M.D. (2003). Process for Making Polytetrafluoroethylene-Aluminum Composites and Product Made. Patent.

[5] Nielson D.B., Truitt R.M., Rasmussen N. (2005). Lower Temperature, Extrudable, High Density Reactive Materials. Patent.

[6] Ames R.G. (2005). Energy release characteristics of impact-initiated energetic materials. MRS Online Proc. Libr..

[7] Ames R.G. A Standardized Evaluation Technique for Reactive Warhead Fragments. Proceedings of the 23rd International Symposium on Ballistics.

[8] Chonowski D.P. (2010). Small Scaled Reactive Materials Combustion Test Facility. Master’s Thesis.

[9] Raftenberg M.N., Mock W., Kirby G.C. (2008). Modeling the Impact Deformation of Rods of a Pressed PTFE/Al Composite Mixture. Int. J. Impact Eng..

[10] Lee R.J., Mock W., Carney J.R., Holt W.H., Pangilinan G.I., Gamache R.M., Boteler J.M., Bohl D.G., Drotar J., Lawrence G.W. (2006). Reactive materials studies. Shock. Compress. Condens. Matter.

[11] Mock W., Drotar J.T. (2007). Effect of Aluminum Particle Size on the Impact Initiation of Pressed PTFE/Al Composite Rods. Shock Compress. Condens. Matter.

[12] Ren H.L., Li W., Ning J.G., Liu Y.B. (2019). The Influence of Initial Defects on Impact Ignition of Aluminum/Polytetrafluoroethylene Reactive Material. Adv. Eng. Mater..

[13] Tang L., Wang H.F., Lu G.C., Zhang H., Ge C. (2021). Mesoscale study on the shock response and initiation behavior of Al-PTFE granular composites. Mater. Des..

[14] Xiao J.G., Wang Z., Nie Z.Y., Tang E.L., Zhang X.P. (2020). Evaluation of Hugoniot parameters for unreacted Al/PTFE reactive materials by modified SHPB test. AIP Adv..

[15] Jiang C.L., Cai S.Y., Mao L., Wang Z.C. (2020). Effect of Porosity on Dynamic Mechanical Properties and Impact Response Characteristics of High Aluminum Content PTFE/Al Energetic Materials. Materials.

[16] Cai J., Walley S.M., Hunt R.J.A., Proud W.G., Nesterenko V.F., Meyers M.A. (2008). High-strain high-strain-rate flow and failure in PTFE/Al/W granular composites. Mater. Sci. Eng. A.

[17] Herbold E.B., Nesterenko V.F., Benson D.J., Cai J., Vecchio K.S., Jiang F., Addiss J.W., Walley S.M., Proud W.G. (2008). Particle size effect on strength, failure, and shock behavior in polytetrafluoroethylene-Al-W granular composite materials. J. Appl. Phys..

[18] Geng B.Q., Wang H.F., Yu Q.B., Zheng Y.F., Ge C. (2020). Bulk Density Homogenization and Impact Initiation Characteristics of Porous PTFE/Al/W Reactive Materials. Materials.

[19] Zhang H., Wang H.F., Ge C. (2020). Characterization of the Dynamic Response and Constitutive Behaviour of PTFE/Al/W Reactive materials. Propellants Explos. Pyrotech..

[20] Glavier L., Taton G., Ducéré J.M., Baijot V., Pinon S., Calais T., Estève A., Rouhani M.D., Rossi C. (2015). Nanoenergetics as pressure generator for nontoxic impact primers: Comparison of Al/Bi_2_O_3_, Al/CuO, Al/MoO_3_ nanothermites and Al/PTFE. Combust. Flame.

[21] Wang L.Y., Jiang J.W., Li M., Men J.B., Wang S.Y. (2021). Improving the damage potential of W-Zr reactive structure material under extreme loading condition. Def. Technol..

[22] Zhou J.Y., Ding L.L., Tang W.H., Ran X.W. (2020). Experimental Study of Mechanical Properties and Impact-Induced Reaction Characteristics of PTFE/Al/CuO Reactive Materials. Materials.

[23] Wang L.L., Hu S.S., Yang L.M., Dong X.L. (2017). Kinetics of Materials.

[24] Johnson G.R., Cook W.H. A constitutive model and data for metals subjected to large strains, high strain rates, and high temperatures. Proceedings of the 7th International Symposium on Ballistics.

[25] Holmquist T.J., Johnson G.R. (1991). Determination of constants and comparison of results for various constitutive models. J. Phys. IV.

